# Optimization of Extraction Conditions and Characterization of Pepsin-Solubilised Collagen from Skin of Giant Croaker (*Nibea japonica*)

**DOI:** 10.3390/md16010029

**Published:** 2018-01-14

**Authors:** Fangmiao Yu, Chuhong Zong, Shujie Jin, Jiawen Zheng, Nan Chen, Ju Huang, Yan Chen, Fangfang Huang, Zuisu Yang, Yunping Tang, Guofang Ding

**Affiliations:** Zhejiang Provincial Engineering Technology Research Center of Marine Biomedical Products, School of Food and Pharmacy, Zhejiang Ocean University, Zhoushan 316022, China; fmyu@zjou.edu.cn (F.Y.); zongchuhong1997@163.com (C.Z.); m18868006087@163.com (S.J.); jwzheng1996@163.com (J.Z.); chennan_0576@163.com (N.C.); qiuqiu20130621@163.com (J.H.); cyancy@zjou.edu.cn (Y.C.); gracegang@126.com (F.H.); abc1967@126.com (Z.Y.); dinggf2007@163.com (G.D.)

**Keywords:** marine collagen, *Nibea japonica*, response surface methodology, optimization, characterization

## Abstract

In the present study, response surface methodology was performed to investigate the effects of extraction parameters on pepsin-solubilised collagen (PSC) from the skin of the giant croaker *Nibea japonica*. The optimum extraction conditions of PSC were as follows: concentration of pepsin was 1389 U/g, solid-liquid ratio was 1:57 and hydrolysis time was 8.67 h. Under these conditions, the extraction yield of PSC was up to 84.85%, which is well agreement with the predict value of 85.03%. The PSC from *Nibea japonica* skin was then characterized as type I collagen by using sodium dodecyl sulfate polyacrylamide gel electrophoresis (SDS-PAGE). The fourier transforms infrared spetroscopy (FTIR) analysis revealed that PSC maintains its triple-helical structure by the hydrogen bond. All PSCs were soluble in the pH range of 1.0–4.0 and decreases in solubility were observed at neutral or alkaline conditions. All PSCs had a decrease in solubility in the presence of sodium chloride, especially with a concentration above 2%. So, the *Nibea japonica* skin could serve as another potential source of collagen.

## 1. Introduction

Collagen is the predominant structural protein in the extracellular matrix of animals, making up about 30% of the total protein content [1,2]. Nowadays, collagen has been widely used in biomedical fields, such as sponges for wound healing [3,4], cornea for ophthalmology [5], hydrogels for articular cartilage [6], scaffolds for bone regeneration [7], and so on. Collagen is also a very attractive ingredient in cosmetics [8]. Furthermore, its hydrolysate (collagen peptide) has also been widely used as function foods or cosmetic additive with its antioxidant activity [9,10]. Collagens used in these fields are commonly extracted from skins or bones from bovine and porcine, while porcine collagens are unacceptable for some religions and bovine collagens are at risk of contamination with prion diseases [11]. Taking into account these limitations, there is the need for preparing safe, high quality collagens from alternative resources.

Recently, recombinant technology has been used to produce human collagen or collagen-like protein, especially expression of hydroxylated collagen [12,13]. Due to its high cost of production and low yield (no more than 2.0 g/L of hydroxylated collagen), it seems not to be a suitable method for industrial production of collagen. Nowadays, collagen extracted from marine fish byproducts has gathered more attention due to non-religious restrictions and safety when compared to other animals [14]. Various marine fish by-products have been used for extracting collagen, such as the skin of *Aluterus monocerous* [2], scales of *Pseudosciaena crocea* [9], skin and bone of *Scomberomorous niphonius* [15], skin and swim bladder of *Lates calcarifer* [16], and so on. The biochemical and functional characteristics of the collagen from different by-products will be different. In addition, the extracted collagen is also used for enzymatic hydrolysis to obtain the bioactive collagen peptides [4,17,18].

Giant croaker, *Nibea japonica* is a carnivorous fish which is cultured and considered as a promising species for marine aquaculture in East Asia because of its high value, fast growth speed, easy receptivity to captivity and the availability of production technology [19,20]. However, collagen from *Nibea japonica* has not been reported and its characterization is also unknown. In this study, pepsin-solubilised collagen (PSC) from *Nibea japonica* skin was extracted and characterized for the first time. So far there is no published work on studies on various extraction conditions on the yield of PSC from *Nibea japonica* skin. As many factors may affect the extraction yield of collagen, response surface methodology (RSM) and Box-Behnken design (BBD) was performed in this study to optimize the extraction conditions for extracting higher yield of PSC. Furthermore, the properties of PSC were also characterized by determining its protein patterns, amino acid composition, fourier transforms infrared spetroscopy (FTIR) spectra and so on.

## 2. Results and Discussion

### 2.1. Single Factor Results

#### 2.1.1. Effect of Enzyme Concentration on the Extraction Yield of PSC

**Figure 1:**
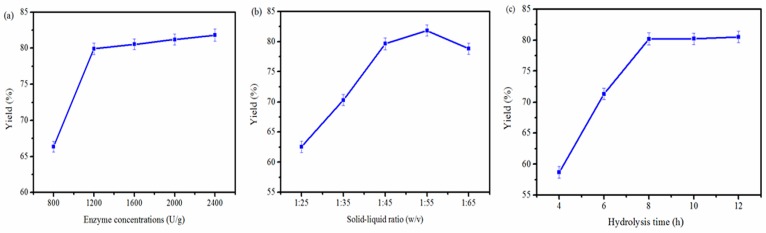
Effects of enzyme concentration (**a**), liquid-solid ratio (**b**) and hydrolysis time (**c**) on extraction yield of collagen from *Nibea japonica* skin.

In the previous studies, collagens were often extracted by using acid extraction and enzymatic extraction [15,16]. However, the acid solubilisation process gives a low yield of collagen. Since pepsin or papain is able to cleave peptides in the telopeptide region of collagen and the helical arrangement can exist in the PSC or papain digested collagen. However, there are some other proteins (above 97.4 kDa) in the papain digested collagen when compared to the PSC [21]. So, pepsin was chosen for extracting collagen in this study. Then, different pepsin concentrations (800, 1200, 1600, 2000 and 2400 U/g) were used to investigate the effect of pepsin concentration on the extracting yield of PSC. The other two extraction parameters were set as follows: solid-liquid ratio was 1:45 and hydrolysis time was 8 h in 0.5 M acetic acid buffer. As shown in Figure 1a, the PSC extraction yield significantly increased from 66.35% to 79.93% when pepsin concentration varied from 800 to 1200 U/g and then slightly increased when pepsin concentration exceeded 1200 U/g. Considering the higher-cost industrial extraction process, the amount of 1200 U/g pepsin was used for further optimization.

#### 2.1.2. Effect of Solid-Liquid Ratio on the Extraction Yield of PSC

Different solid-liquid ratio (1:25, 1:35, 1:45, 1:55 and 1:65) was used to study the effect of solid-liquid ratio on the extraction yield of PSC. The other two extraction parameters were set as follows: pepsin concentration was 1200 U/g and hydrolysis time was 8 h in 0.5 M acetic acid buffer. As shown in Figure 1b, the extraction yield of PSC was significantly increased with increasing liquid-solid ratio between 1:25 and 1:55. So, the solid-liquid ratio of 1:55 was selected for next optimization.

#### 2.1.3. Effect of Hydrolysis Time on the Extraction Yield of PSC

To check the effect of hydrolysis time on the extraction yield of PSC, different hydrolysis time (4, 6, 8, 10 and 12 h) was carried out in this study. The other two extraction parameters were set as follows: pepsin concentration was 1200 U/g and solid-liquid ratio was 1:55 in 0.5 M acetic acid buffer. As shown in Figure 1c, the extraction yield of PSC was significantly increased with hydrolysis time between 4 and 8 h. Therefore, the hydrolysis time of 8 h was selected for the next optimization.

### 2.2. Optimization of Extraction Parameters of PSC Using RSM

#### 2.2.1. Response Surface Analysis

After screening, RSM using BBD was used to obtain the optimal levels of the three above factors that significantly affected the yield of PSC. The experimental design and results are shown in Table 1 On the basis of the regression analysis of the data in Table 1, the effects of these three factors on the extraction yield of PSC were predicted by using a second-order polynomial function as follows: *Y* = 83.73 + 3.53*X*_1_ + 1.47*X*_2_ + 1.78*X*_3_ − 0.11*X*_1_*X*_2_ − 0.59*X*_1_*X*_3_ − 0.80*X*_2_*X*_3_ − 3.68*X*_1_^2^ − 2.03*X*_2_^2^ − 1.89*X*_3_^2^ (where *Y* was the extraction yield of PSC, and *X*_1_, *X*_2_, *X*_3_ were the pepsin concentration, solid-liquid ratio and hydrolysis time, respectively).

In order to determine the significance of the quadratic model, the analysis of variance (ANOVA) was performed and the results are shown in Table 2. As suggested by the model *F* value and a low probability value (*p* = 0.0001), it was obvious that the model was highly significant. The lack of fit *F* value in this model was about 6.13 and it suggested that the lack of fit was not significant relative to the pure error. The determination coefficient (*R*^2^ = 0.9920) by ANOVA of this model and the adjusted determination coefficient (Adj *R*^2^ = 0.9777) also indicated that the model was highly significant. So, this model was selected in this study for optimizing.

**Figure 2:**
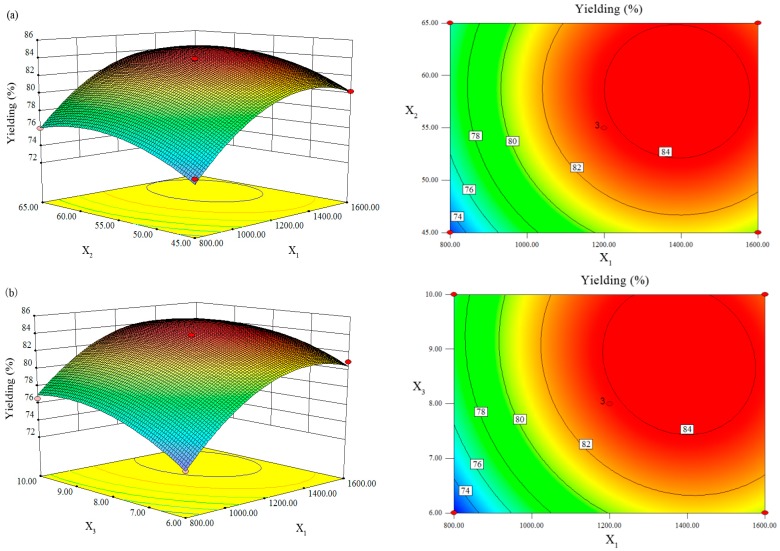

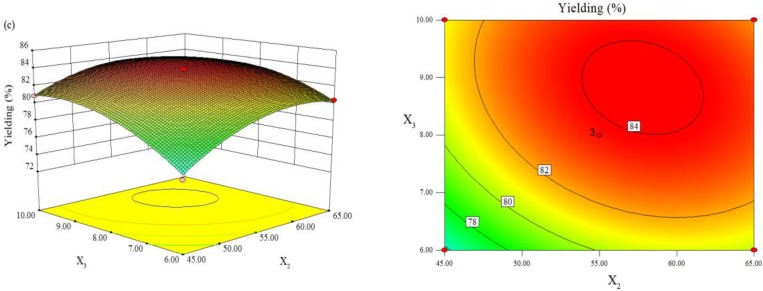
Three-dimensional response surface plots (**left**) and two-dimensional contour plots (**right**) showing the effects of (**a**) enzyme concentration (*X*_1_) vs. liquid-solid ratio (*X*_2_), (**b**) enzyme concentration (*X*_1_) vs. hydrolysis time (*X*_3_) and (**c**) liquid-solid ratio (*X*_2_) vs. hydrolysis time (*X*_3_) on extraction yield of collagen from *Nibea japonica* skin.

**Figure 3:**
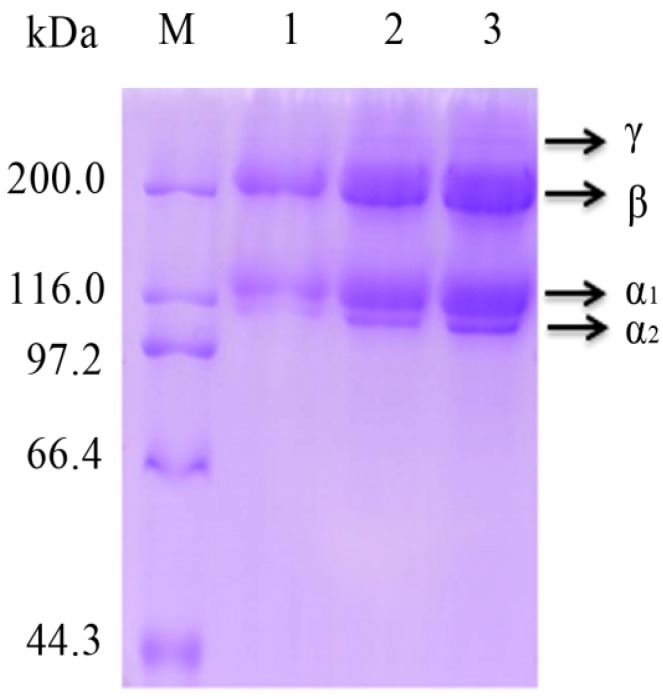
Sodium dodecyl sulfate polyacrylamide gel electrophoresis (SDS-PAGE) analysis of PSC from *Nibea japonica* skin. M: Protein molecular weight marker; Lane 1–3: Purified PSC from *Nibea japonica* skin.

Furthermore, three-dimensional response surfaces and contour plots were generated from the model equation to visualize the relationship between the extraction yield of PSC and extraction factors (Figure 2). It also show the optimal levels of each component required for the extraction of PSC (Figure 3). These three-dimensional response surfaces and contour plots provided a visual interpretation of the mutual interactions between two factors. The maximum predicted yield of PSC was 85.03% under the following conditions: concentration of enzyme was 1389 U/g, solid-liquid ratio was 1:57 and hydrolysis time was 8.67 h.

#### 2.2.2. Validation of the Models

Three additional experiments were performed in order to verify the predicted yield under the optimal extraction conditions. The mean value of PSC yield was 84.85%, which was in excellent agreement with the predicted value, under the similar conditions.

### 2.3. SDS-PAGE Analysis

The protein patterns of PSC from *Nibea japonica* skin were analyzed by SDS-PAGE (Figure 3). As shown in Figure 3, PSC from *Nibea japonica* skin consisted of two α_1_-chains and one α_2_-chain. The β and γ chains as well as the cross-linked constituents were also observed in this study (Figure 3). PSC extracted from *Nibea japonica* skin may have the structure of (α_1_)_2_α_2_, which was classified as Type I collagen. Our results were consistent with the collagens from other marine fish skins, such as PSC from *Aluterus monocerous* [2], PSC from *Scomberomorous niphonius* [15] and PSC from *Istiophorus platypterus* [22].

### 2.4. Amino Acid Composition of PSC

The amino acid composition of PSC from *Nibea japonica* skin was determined and the results are shown in Table 3 and compared with collagen from calf skin, type I collagen from porcine skin and human [23,24,25]. The most abundant amino acids found in PSC from *Nibea japonica* skin were glycine (Gly), alanine (Ala), proline (Pro) and hydroxyproline (Hyp). In this study, Gly was found to be the major amino acid in PSC (348 residues/1000 residues), the result is accordance with the (Gly-Xaa-Yaa) n repeat structure in all collagen molecules. It is known that the Xaa and Yaa positions can be occupied by any other amino acid, but the most common residue for Xaa is Pro and for Yaa is Hyp [26], forming the most common triplet repeats that found in most collagens (Gly-Pro-Hyp) n [22]. The Pro and Hyp contents of the PSC was 116 residues/1000 residues and 75 residues/1000 residues, respectively, which is similar to that of PSC from skin of *Aluterus monocerous* [2]. The rate of proline hydroxylation was about 39.3% for PSC from *Nibea japonica* skin. There were no tryptophan and cysteine residues in the PSC from *Nibea japonica* skin.

### 2.5. UV-Visible Spectroscopy

**Figure 4:**
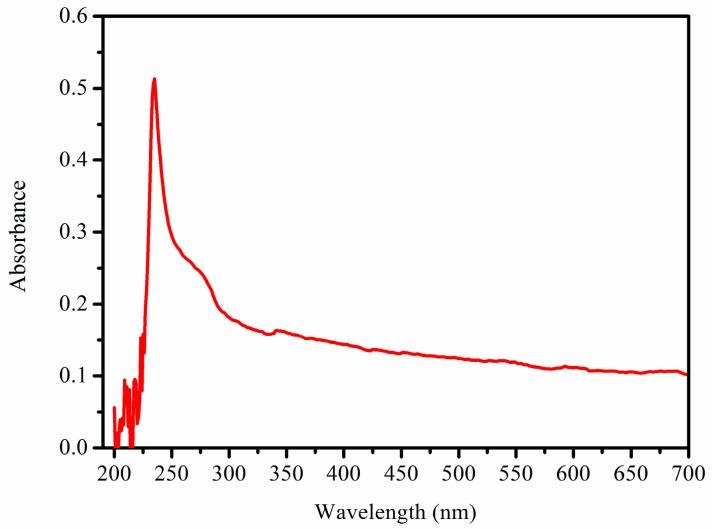
UV-visible spectroscopy of PSC from *Nibea japonica* skin.

It is known that collagen has a single absorption peak at 230 nm because of its triple helical structure, so UV-visible spectroscopy of collagen can be used to evaluate its purity [14,27]. As shown in Figure 4, PSC extracted from *Nibea japonica* skin showed a single absorption peak at 230 nm. Our result was similar to the collagen that has been isolated from other fish species. It is also necessary to point out that no any other obvious peaks were found at 280–300 nm while other proteins usually have absorption peaks at 280 nm. This is because the tyrosine content in collagen was very low. Finally, the UV-visible spectroscopy of PSC indicated that the extracted proteins using pepsin extraction was collagen and it also shown that pepsin extraction was the efficient methods to obtain purity collagens.

### 2.6. Fourier Transforms Infrared Spetroscopy (FTIR) Analysis

**Figure 5:**
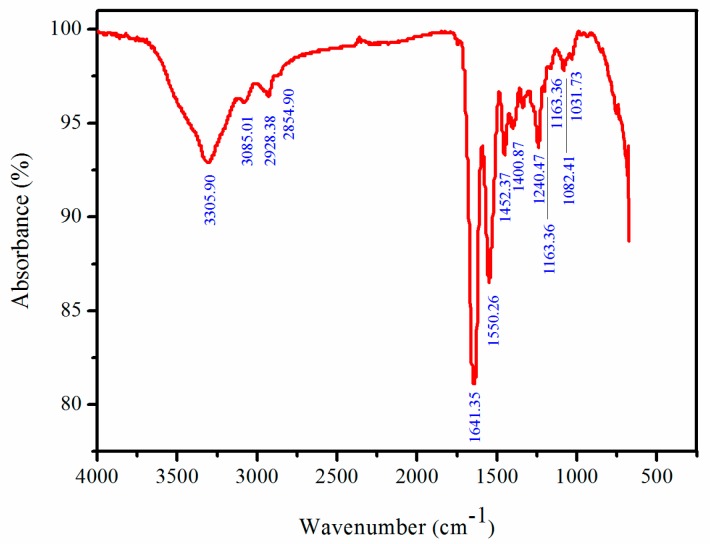
FTIR analysis of PSC from *Nibea japonica* skin.

The FTIR spectra of PSC from *Nibea japonica* skin is shown in Figure 5. These peaks correspond to five main amide bonds (amide A, B, I, II and III). The amide A bands of PSC was measured at 3305.90 cm^−1^. The value is associated with N-H stretching frequency and indicate the presence of hydrogen bonds. The free N-H frequency vibration occurs at 3400–3440 cm^−1^ and shifts lower to 3300 cm^−1^ [28]. The amide B band of PSC was measured at 2928.38 cm^−1^, which was consistent with asymmetrical stretch of CH_2_ [29]. The amide I band of PSC was detected at 1641.35 cm^−1^, fitting well with the range of 1600–1700 cm^−1^ for general amide I band position. The amide II band of PSC was measured at 1550.26 cm^−1^, fitting well with the range of amide II band position (1550–1600 cm^−1^). Finally, the amide III band of PSC was measured at 1240.47 cm^−1^, which indicated the helical arrangement existed in the PSC from *Nibea japonica* skin [29,30].

### 2.7. Effects of pH and Sodium Chloride on PSC Solubility

**Figure 6:**
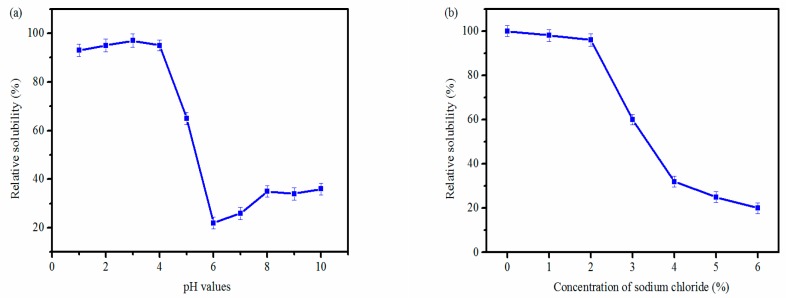
Effects of pH (**a**) and sodium chloride (**b**) on PSC solubility.

The effects of pH and sodium chloride on the solubility of PSC from *Nibea japonica* skin were also investigated in the present study. As shown in Figure 6a, the PSC was dissolved in the acidic pH range of 1.0–4.0. The decrease in solubility was observed in the pH range of 5.0–7.0 and the dissolved protein was found to be deposited in this pH range. However, the slight increase in solubility was shown in the pH range of 8.0–10.0. Our results were consistent with the collagens from the skin of *Spanish mackerel* [15], bone and skin of *Hypophthalmichthys molitrix* [14] and skin of *Ictalurus punctatus* [31]. As shown in Figure 6b, all PSCs had a slight decrease in solubility with the concentrations of sodium chloride lower than 2% and drastic decrease was observed with the concentrations of sodium chloride higher than 2%. Similar reports were reported from the skin of *Ictalurus punctatus* [31] and skin of *Aluterus monocerous* [2]. 

## 3. Materials and Methods

### 3.1. Materials and Chemical Reagent

*Nibea japonica* skins were provided by Zhejiang Marine Fisheries Research Institution (Zhoushan, China). The age of these fish was about one year and the average weight is about 0.4–0.5 kg. The fish skins were thawed at 4 °C and the residues under skins were removed. The cleaned fish skins were cut into small pieces and then stored at −20 °C until used for extracting of collagen. Pepsin was purchased from YTHX Biotechnology Co., Ltd. (Beijing, China). l-hydroxyproline and chloramine T trihydrate were purchased from Aladdin (Shanghai, China). All other reagents used were analytical grade.

### 3.2. Extraction of PSC from Nibea japonica Skin

The PSC from *Nibea japonica* skin was extracted according to the previous methods with slight modification [11,32]. Ten grams of fish skins were weighed precisely and in order to remove other proteins, the fish skins were then treated with 10 volumes (*v*/*w*) of 0.1 M NaOH for 24 h at 4 °C with continuous stirring. The alkali-treated fish skins were neutralized by washing repeatedly with pre-cooling distilled water and extracted by 1200 U/g pepsin in a ratio of 1:55 (*w*/*v*) for 10 h at 4 °C in 0.5 M acetic acid. The extractions were then centrifuged at 12,000 rpm for 10 min at 4 °C and the supernatants were collected. The supernatants were dialyzed against cold distilled water until the neutral pH was reached by using a dialysis bag with molecular weight cut-off of 25 kDa at 4 °C with a gentle stirring. The final solution was then lyophilized using d freeze dryer (ALPHA 1-2 LD plus, Christ, Germany). The Hyp content in the PSC or fish skins was calculated according to the previous study [33] and the extraction yield of PSC was calculated using the equation as follows:

(1) $$\mathrm{PSC}\;\mathrm{extraction}\;\mathrm{yield}\;(\%)=\frac{\mathrm{Hydroxyproline}\;\mathrm{content}\;\mathrm{in}\;\mathrm{PSC}}{\mathrm{Hydroxyproline}\;\mathrm{content}\;\mathrm{in}\;\mathrm{fish}\;\mathrm{skin}}\times 100\%$$

### 3.3. Experimental Design and Statistical Analysis

Single factor experiments were carried out for establishing the preliminary range of the extraction variables, such as pepsin concentration, solid-liquid ratio and hydrolysis time. Then, RSM and BBD were applied to optimize the three extraction parameters for improving the yield of PSC from *Nibea japonica* skin. The range and levels of the variables investigated in the present study were given in Table 4.

RSM with BBD was performed to obtain the optimum conditions for PSC extraction. For statistical calculations, the factors were coded according the equation as follows:

(2) $$\chi_{i}=\frac{X_{i}-X_{o}}{\Delta X}$$

where, χ*_i_* is the coded value of the independent factor, *X_i_* is the actual value of the independent factor, *X*_o_ is the actual value of *X_i_* at the center point and Δ*X* is the step change value. As shown in Table 2, the Box-Behnken design in the experiment design consists of 15 experimental points and the data from experiment design were explained by multiple regressions to fit the second-order polynomial equation as follows:

(3) $$Y=\beta_{o}+\sum \beta_{i}X_{i}+\sum \beta_{ij}X_{i}X_{j}+\sum \beta_{ii}X_{i}X_{i}$$

where, *Y* is the dependent variable (PSC yield, %); β_o_ is the intercept term, β*_i_* is the linear regression coefficient, β*_ii_* is the aquared coefficient and β*_ij_* is the interaction coefficient. *X_i_* and *X_j_* are levels of the independent variables. Each experiment design was determined in triplicate and the data were analyzed using the software Design-Expert 8.0.5 (State-Ease Inc., Minneapolis, MN, USA).

### 3.4. SDS-PAGE Analysis

The PSC samples from *Nibea japonica* skin were then analyzed by using SDS-PAGE according to the method described by Tang et al. [34]. The PSC samples were firstly dissolved in 0.5 M acetic acid and mixed with the loading buffer. Electrophoresis was performed on 7.5% gels and high protein molecular weight marker (Takara, Dalian, China) was used to estimate the molecular weight of PSC.

### 3.5. Amino Acid Composition of PSC

The lyophilized PSC samples were hydrolyzed with 6 M HCl at 110 °C for 24 h without oxygen and then vaporized. The hydrolysates were then analyzed by using a Hitachi amino acid analyser L-8800 (Hitachi, Tokyo, Japan). The content of Hyp was accurately measured according to the protocol described by Tang et al. [1].

### 3.6. UV-Visible Spectroscopy of PSC

The UV-visible spectrum of PSC from *Nibea japonica* skin was determined by a Shimadzu spectrophotometer (Shimadzu, Kyoto, Japan). The lyophilized PSC samples were dissolved in 0.5 M acetic acid and then centrifuged at 12,000 rpm for 10 min at 4 °C. The absorbance of the supernatant was measured at different wavelengths (from 200 nm to 700 nm) to get its UV-visible spectrum. 

### 3.7. FTIR Spectra of PSC 

FTIR spectra of lyophilized PSC samples were measured on a Bruker Tensor 27 FTIR spectrometer (Bruker, Rheinstetten, Germany) using the method described by previous studies. The spectra were produced with a wavelengths range from 4000 to 450 cm^−1^ at a resolution of 1 cm^−1^ for a single scan.

### 3.8. Effects of pH and Sodium Chloride on PSC Solubility

Lyophilized PSC samples (3 mg/mL) were first dissolved in 0.5 M acetic acid. Then, PSC solution (8 mL) was transferred to a centrifuge tube (15 mL) and the pH was adjusted with 6 N HCl or 6 N NaOH to get the final pH ranging from 1.0 to 10.0 and constant-volumed to 10 mL by deionized water. The mixtures were centrifuged at 12,000 rpm for 10 min at 4 °C and the protein content in the supernatant was measured by the Bradford method. 

The effect of sodium chloride on PSC solubility was determined as follows: PSC (6 mg/mL) was dissolved in 0.5 M acetic acid and 5 mL of this solution was added with 5 mL sodium chloride (in 0.5 M acetic acid) with a series of concentrations (0, 2, 4, 6, 8, 10 and 12%) to the final concentrations of 0, 1, 2, 3, 4, 5 and 6%. The mixtures were then stirred at 4 °C for 30 min and then centrifuged at 12,000 rpm for 10 min at 4 °C. The protein content in the supernatant was measured as described above.

## 4. Conclusions

In the present study, RSM was used to optimize the extraction process of PSC from *Nibea japonica* skin. The pepsin concentration of 1389 U/g, solid-liquid ratio of 1:57 and hydrolysis time of 8.67 h was found to be optimal for PSC extraction; giving a yield of 84.85%. The extracted PSC was then characterized as type I collagen using SDS-PAGE electrophoresis, and the FTIR analysis also revealed that PSC maintains its triple-helical structure. All PSCs were soluble in the pH range of 1.0–4.0 and decreases in solubility were observed at neutral or alkaline conditions. All PSCs had a decrease in solubility in the presence of sodium chloride, especially with a concentration above 2%. Further study will be performed to investigate whether this collagen can be used in biomedical applications and other fields.

## Figures and Tables

**Table 1:** The Box-Behnken design and the response for the extraction yield of pepsin-solubilised collagen (PSC).

Runs	Enzyme Concentration (*X*_1_)	Solid-Liquid Ratio (*X*_2_)	Hydrolysis Time (*X*_3_)	PSC Yield (%) (*Y*)
1	0	0	0	83.88
2	0	1	−1	81.38
3	−1	1	0	76.01
4	0	1	1	82.78
5	1	1	0	82.36
6	0	0	0	83.39
7	−1	0	−1	75.97
8	0	−1	−1	75.24
9	0	−1	1	80.85
10	1	0	−1	80.95
11	1	0	1	82.91
12	1	−1	0	80.25
13	0	0	0	83.91
14	−1	0	1	76.53
15	−1	−1	0	74.44

**Table 2:** Analysis of variance of regression model.

Source	Sum of Squares	df	Mean Square	*F* Value	*p* Value
Model	216.13	9	24.01	69.10	0.0001
*X*_1_	99.83	1	99.83	287.24	<0.0001
*X*_2_	17.26	1	17.26	49.66	0.0009
*X*_3_	25.45	1	25.45	73.24	0.0004
*X*_1_*X*_2_	0.053	1	0.053	0.15	0.7125
*X*_1_*X*_3_	1.37	1	1.37	3.94	0.1040
*X*_2_*X*_3_	2.58	1	2.58	7.41	0.0417
*X*_1_^2^	50.13	1	50.13	144.23	<0.0001
*X*_2_^2^	15.17	1	15.17	43.65	0.0012
*X*_3_^2^	13.15	1	13.15	37.83	0.0017
Residual	1.74	5	0.35		
Lack of fit	1.57	3	0.52	6.13	0.1435
Pure Error	0.17	2	0.085		
Cor Total	217.86	14			
*R*^2^					0.9526
Adj *R*^2^					0.9777

**Table 3:** Amino acid compositions of PSC from *Nibea japonica* skin (results are expressed as residues/1000 residues).

Amino Acid	*Nibea japonica* Skin PSC	Calf Skin Collagen [23]	Type I Collagen of Porcine Skin [24]	Type I Collagen of Human [25]
Aspartic acid	43	45	44	43
Threonine	20	18	16	17
Serine	29	33	33	33
Glutamic acid	73	75	72	71
Glycine	348	330	341	335
Alanine	128	119	115	111
Cysteine	0	0	0	0
Valine	19	21	22	26
Methionine	10	6	6	6
Isoleucine	9	11	10	9
Leucine	25	23	22	23
Tyrosine	3	3	1	2
Phenylalanine	6	3	12	12
Histidine	8	5	5	6
Lysine	30	26	27	23
Arginine	51	50	48	50
Proline	116	121	123	120
Hydroxyproline	75	94	97	103
Imino acid	191	215	220	223

**Table 4:** Independent factors and their levels used in the response surface design.

Independent Factors	Symbol	Level of Factor		
		−1	0	1
Enzyme concentration (U/g)	*X*_1_	800	1200	1600
Solid-liquid ratio (*v*/*w*)	*X*_2_	1:45	1:55	1:65
Hydrolysis time (h)	*X*_3_	6	8	10
